# Manassantin B shows antiviral activity against coxsackievirus B3 infection by activation of the STING/TBK-1/IRF3 signalling pathway

**DOI:** 10.1038/s41598-019-45868-8

**Published:** 2019-06-28

**Authors:** Jae-Hyoung Song, Jae-Hee Ahn, Seong-Ryeol Kim, Sungchan Cho, Eun-Hye Hong, Bo-Eun Kwon, Dong-eun Kim, Miri Choi, Hwa-Jung Choi, Younggil Cha, Sun-Young Chang, Hyun-Jeong Ko

**Affiliations:** 1 College of Pharmacy, Kangwon National University, Chuncheon, South Korea; 2 Research Institute of Pharmaceutical Science and Technology (RIPST), College of Pharmacy, Ajou University, Suwon, South Korea; 3 Anticancer Agent Research Center, Korea Research Institute of Bioscience & Biotechnology, Ochang, South Korea; 4 Department of Beauty Science, Kwangju Women’s University, Gwangju, South Korea

**Keywords:** Viral infection, Antivirals

## Abstract

Coxsackievirus B3 (CVB3) is an important human pathogen associated with the development of acute pancreatitis, myocarditis, and type 1 diabetes. Currently, no vaccines or antiviral therapeutics are approved for the prevention and treatment of CVB3 infection. We found that *Saururus chinensis* Baill extract showed critical antiviral activity against CVB3 infection *in vitro*. Further, manassantin B inhibited replication of CVB3 and suppressed CVB3 VP1 protein expression *in vitro*. Additionally, oral administration of manassantin B in mice attenuated CVB3 infection-associated symptoms by reducing systemic production of inflammatory cytokines and chemokines including TNF-α, IL-6, IFN-γ, CCL2, and CXCL-1. We found that the antiviral activity of manassantin B is associated with increased levels of mitochondrial ROS (mROS). Inhibition of mROS generation attenuated the antiviral activity of manassantin B *in vitro*. Interestingly, we found that manassantin B also induced cytosolic release of mitochondrial DNA based on cytochrome C oxidase DNA levels. We further confirmed that STING and IRF-3 expression and STING and TBK-1 phosphorylation were increased by manassantin B treatment in CVB3-infected cells. Collectively, these results suggest that manassantin B exerts antiviral activity against CVB3 through activation of the STING/TKB-1/IRF3 antiviral pathway and increased production of mROS.

## Introduction

The family *Picornaviridae* is currently divided into 35 genera, three of which are important causes of human infectious diseases^1^. Coxsackievirus B3 (CVB3) is included in the genus *Enterovirus* within the family *Picornaviridae* and is a major causative agent of cardiac muscle infection^2–4^. In addition, CVB3 occasionally causes chronic pancreatic inflammatory diseases resulting in insulin-dependent type I diabetes mellitus and idiopathic chronic pancreatitis^4–6^. However, specific clinical therapy is not presently available for this virus type, although a number of antiviral candidates are under development^7^.

Medicinal plants—with often manifold targets, less severe side effects, low potential to cause drug tolerance, and low cost—are increasingly considered suitable alternative sources of antiviral candidates^8–12^. *Saururus chinensis* Baill, a well-known medicinal plant of China and South Korea, has been traditionally used for the treatment of beriberi, hypertension, pneumonia, oedema, jaundice, leproma, and gonorrhoea^13,14^. *Saururus* chemical studies have revealed the presence of more than 20 lignans^15^, some of which exhibit neuroleptic^16^, hepatoprotective^17^, and antifeedant activities^18^. Additionally, *S. chinensis* Baill was reported to protect against sepsis^15^, cell adhesion^19^, inflammation^20,21^, and hypercholesterolemia^22^. Other types of compounds found in *S. chinensis* Baill include aristolactams, flavonoids, anthraquinones, and fruanoditerpenes^23,24^.

In the current study, we found that *S. chinensis* Baill extract exerted significant antiviral activity against CVB3 infection in Vero cells and revealed manassantin B (Man B) as one of the antiviral components isolated from the ethyl acetate fraction of the *S. chinensis* Baill extract. Man B protected mice from systemic infection of CVB3 and reduced CVB3-mediated inflammatory cytokine production. Man B exerted antiviral activity against CVB3 through activation of the STING/TBK-1/IRF3 signalling pathway as well as increased production of mitochondrial ROS (mROS). This study may shed light on a medicinal plant whose extract or active compounds may be used clinically for the effective treatment of enterovirus infection.

## Results

### Man B from *S. chinensis* Baill extract showed antiviral activity against CVB3 *in vitro*

We found that the *S. chinensis* Baill extract showed antiviral activity against CVB3 *in vitro* during screening of antiviral candidates from medicinal plants. Both the hexane and ethyl acetate fraction of the methanol extract of *S. chinensis* Baill showed strong antiviral activity (Supplementary Table S1).

The ethyl acetate fraction was further fractionated using C18 column chromatography; Fr.10 showed the highest antiviral activity (Supplementary Table S2). We isolated one active compound from Fr.10, which was identified as Man B using UV spectroscopic, EI-MS, ^1^H-NMR, and ^13^C-NMR analysis (Supplementary Fig. S1). Man B is a lignin compound known to exist in *S. chinensis* Baill^25^. The interpretations of proton and carbon signals were consistent with those of Seo *et al*. (2009). Man B is a yellow amorphous powder characterised as follows: ESI-MS (70 eV), m/z (% relative intensity): 739.9 [M + Na]^+^, ^1^H NMR (DMSO-d_6_, 400 MHz), and ^13^C NMR (DMSO-d_6_, 100 MHz) (Supplementary Table S3).

### Man B inhibited the replication of CVB3 in Vero cells

**Figure 1:**
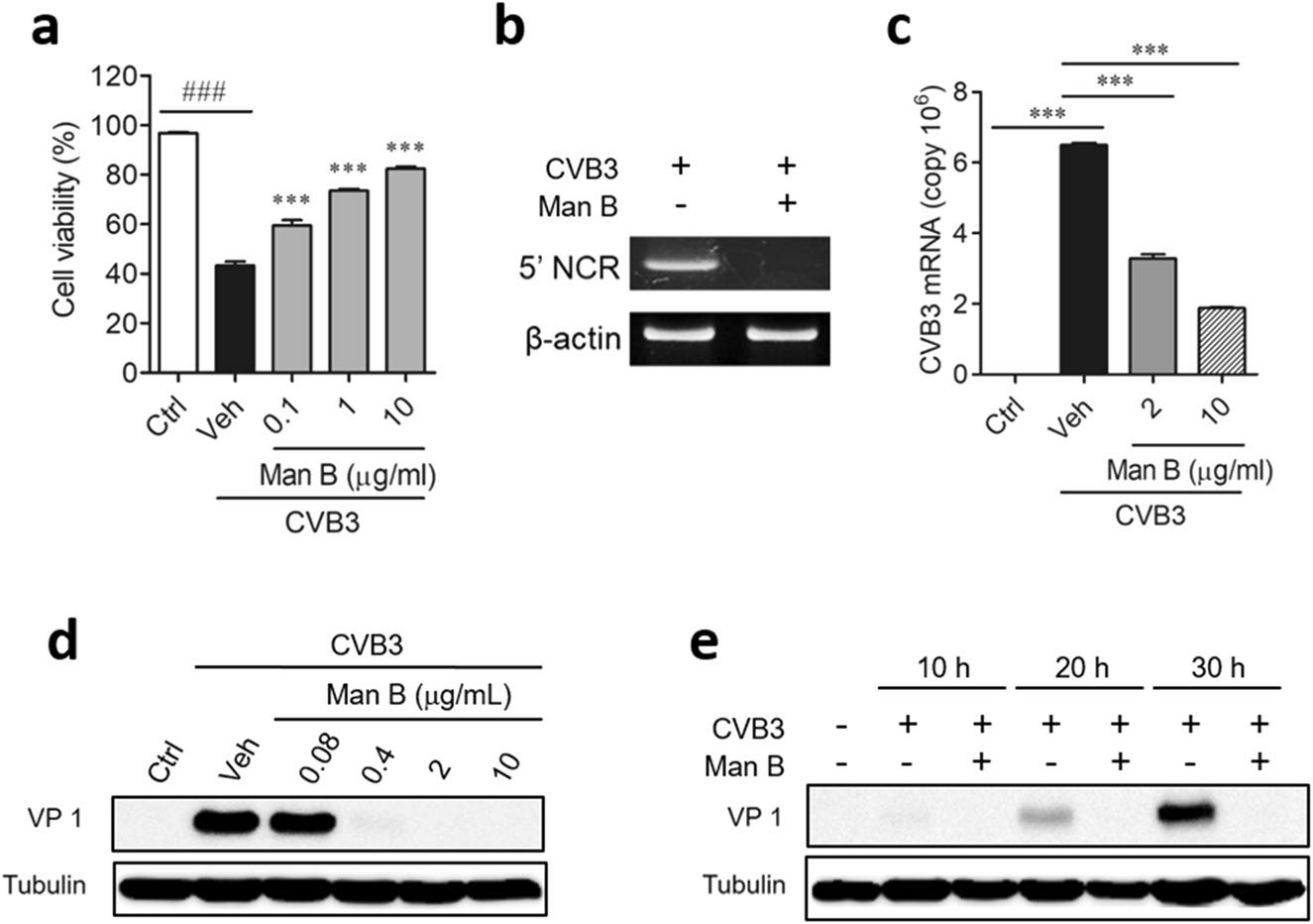
Antiviral activity of manassantin B against coxsackievirus B3 *in vitro*. (**a**) Vero cells were infected with coxsackievirus B3 **(**CVB3) at 50% cell culture infective dose (CCID_50_) and treated with manassantin B (Man B). The cytopathic effect on virus-infected Vero cells was analysed 2 days after infection. The antiviral activity was calculated based on cell viability. (**b**) Replication of CVB3 in Vero cells at 48 h after infection with CVB3 in the presence of Man B (10 µg/mL) was detected by RT-PCR of the CVB3 genome. (**c**) Quantitative real-time PCR was conducted to measure the copy number of CVB3 viral RNA in Vero cells after treatment with Man B (2 and 10 μg/mL). (**d**) Western blotting was performed to determine the effect of different concentrations of Man B on the production of CVB3 VP1 protein. Vero cells (1 × 10^6^) were seeded in a 6-well plate and incubated overnight. Cells were infected with CVB3 at an MOI of 0.8 and treated with 0.08, 0.4, 2, and 10 μg/mL of Man B. After 48 h, cells were harvested. (**e**) To evaluate the antiviral activity of Man B at multiple time points, cells were harvested at 10, 20, and 30 h after CVB3 infection and treatment with 2 µg/mL of Man B. Thirty micrograms of total cellular proteins from CVB3-infected Vero cells was electrophoresed and subjected to western blot. α-Tubulin was used as a loading control for each set of samples. The samples were derived from the same experiment, and the gels/blots were processed in parallel. Results are shown as means ± SEM of 3 independent experiments. ^*###*^*P* < 0.001 for comparison with non-infected control group (Ctrl); ***P < 0.001 for comparison with CVB3-infected vehicle group (Veh), Bonferroni’s multiple comparison test (ANOVA).

To confirm the antiviral activity of Man B against CVB3, the effect of Man B on the viability of Vero cells infected with CVB3 was evaluated. Man B significantly blocked the CVB3-mediated cytotoxicity of Vero cells (Fig. 1a). In addition, CVB3 replication in Vero cells was inhibited by Man B as determined by RT-PCR (Fig. 1b) and quantitative real-time RT-PCR (Fig. 1c, Supplementary Fig. S2) of the CVB3 RNA genome. We also confirmed that CVB3 VP1 protein expression was completely suppressed by 0.4 μg/mL Man B treatment through western blot analysis (Fig. 1d) and that Man B strongly suppressed viral replication and capsid protein production at the early stage of the virus life cycle (Fig. 1e). Therefore, we confirmed the antiviral activity of Man B against CVB3 infection *in vitro*. To determine whether the antiviral activities of Man B were restricted to CVB3, we investigated its antiviral activity against other coxsackieviruses including CVB1, CVB2, CVB4, CVB5, and CVB6 (Supplementary Fig. S3). Interestingly, Man B displayed significant antiviral activity against all tested group B coxsackieviruses with 50% inhibitory concentration (IC_50_) values of 0.88–8.20 μg/mL (Supplementary Table S4).

To evaluate whether Man B inhibits CVB3 replication, we conducted a time-of-drug addition assay. We found that the addition of Man B at 8 h after CVB3 infection did not show antiviral activity, suggesting that Man B plays an inhibitory role in the early period of CVB3 infection (Supplementary Fig. S4). To determine whether Man B directly affects viral protein translation, we performed an internal ribosome entry site (IRES) binding inhibition assay with an IRES dual reporter system^26^. As a result, the ratio of Renilla luciferase activity, which reflects Cap-dependent translation to firefly luciferase activity, which reflects IRES translation was not significantly different between the vehicle- and Man B-treated groups. Thus, Man B did not appear to directly inhibit the viral IRES-dependent translation (Supplementary Fig. S5).

### Man B reduced pro-inflammatory cytokines and chemokines in the serum of CVB3-infected mice

**Figure 2:**
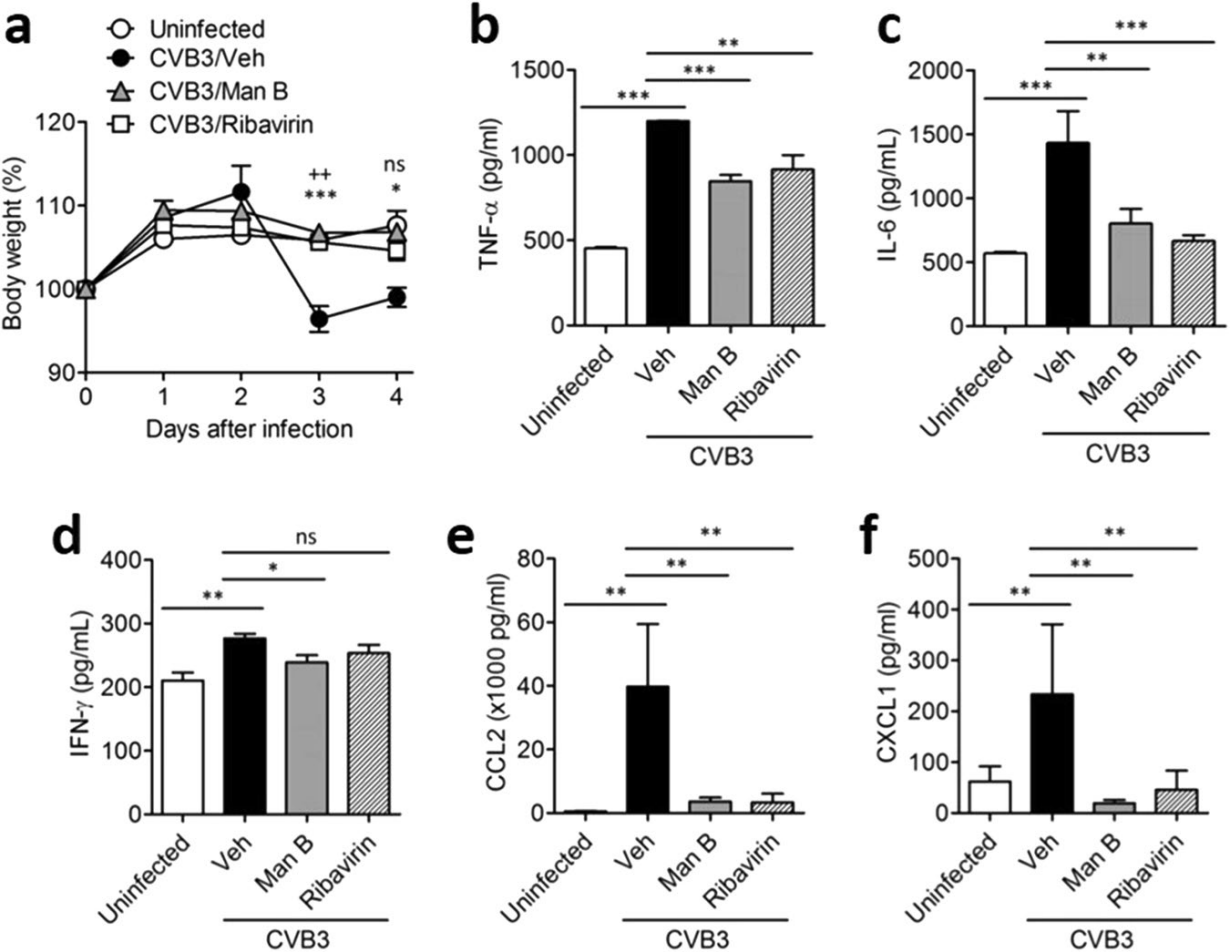
Antiviral activity of manassantin B against coxsackievirus B3 *in vivo*. BALB/c mice (n = 5/group) were intraperitoneally infected with a 1 × 10^6^ pfu/100 µL of coxsackievirus B3 (CVB3) and administered manassantin B (Man B) (2.5 mg/kg), ribavirin (10 mg/kg), or vehicle (Veh) (0.5% carboxymethyl cellulose). (**a**) Body weight was measured for 5 days. Results are shown as means ± SEM of 3 independent experiments. ^++^*P* < 0.01 for comparison with CVB3/Veh and CVB3/Ribavirin; *P < 0.05, ***P < 0.001 for comparison with CVB3/Veh and CVB3/Man B, Bonferroni’s multiple comparison test (ANOVA). Mice were infected with 1 × 10^6^ pfu/100 µL of CVB3. After 5 days of infection, the level of chemokines and cytokines was analysed by ELISA. Levels of (**b**) TNF-α, (**c**) IL-6, (**d**) IFN-γ, (**e**) CCL2, and (**f**) CXCL-1 in serum isolated from control mice, vehicle-treated, CVB3-infected mice (Veh), manassantin B-treated, CVB3-infected mice, or ribavirin-treated, CVB3-infected mice. Data represent the mean values of five mice per group. Results are shown as means ± SEM of 3 independent experiments. *P < 0.05, **P < 0.01, ***P < 0.001, Bonferroni’s multiple comparison test (ANOVA).

To assess whether Man B elicited antiviral activity *in vivo*, we adopted the pancreatic infection model of CVB3 as we previously reported^27^. Intraperitoneal injection of 1 × 10^6^ pfu CVB3 resulted in body weight loss of BALB/c mice, whereas daily injection of 2.5 mg/kg Man B or 10 mg/kg ribavirin for 4 days prevented body weight loss caused by CVB3 infection (Fig. 2a). Likewise, the level of serum cytokines and chemokines including TNF-α, IL-6, IFN-γ, CCL2, and CXCL-1, which increased after 5 days post CVB3 infection, was significantly reduced by treatment with Man B or ribavirin (Fig. 2b–f). Collectively, these results suggested that Man B ameliorated CVB3 infection-associated symptoms similarly to ribavirin.

### Man B induced mitochondrial damage with mROS generation

**Figure 3:**
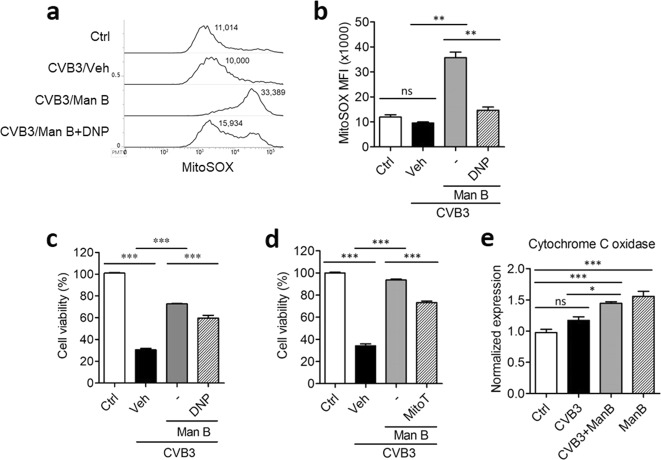
Manassantin B induced mitochondrial ROS and release of mitochondrial DNA into the cytosol. (**a**) Coxsackievirus B3 (CVB3)-infected Vero cells were treated with manassantin B (Man B) with or without 20 µM 2,4-dinitrophenol (DNP). Harvested cells were stained with 5 µM of MitoSOX reagent and analysed by fluorescence-activated cell sorting (FACS) to detect reduction in mitochondrial ROS. (**b**) The mean MitoSOX fluorescence index (MFI) was measured. (**c**) 50 µM of DNP and (**d**) 50 µM of mitoTempo (MitoT) were added to Man B-treated and CVB3-infected cells. (**e**) Cytosolic mitochondrial DNA was detected by PCR. Ctrl, control; Veh, vehicle. Data are presented as means ± SEM of 5 independent experiments. *P < 0.05, **P < 0.01, ***P < 0.001, Bonferroni’s multiple comparison test (ANOVA).

Treatment of CVB3-infected Vero cells with Man B considerably increased the levels of mROS. Addition of the mitochondrial uncoupling agent 2,4-dinitrophenol (DNP) reduced the generation of mROS following Man B treatment (Fig. 3a,b). In addition, treatment of CVB3-infected Vero cells with 50 μM of DNP, a mitochondrial electron transport chain uncoupler, or mitoTempo, a mitochondria-targeted antioxidant, significantly inhibited the antiviral activity of Man B, albeit not completely (Fig. 3c,d).

We next assessed whether Man B also induced cytosolic release of mitochondrial DNA (mtDNA), since mtDNA was recently found to trigger innate antiviral signalling^28^. For this, cytochrome C oxidase DNA was amplified from genomic DNA of the cytosolic fraction. As a result, Man B treatment increased cytosolic cytochrome C oxidase DNA release (Fig. 3d).

### Mitochondrial electron transport chain complex V inhibitor showed antiviral activity against CVB3 *in vitro*

**Figure 4:**
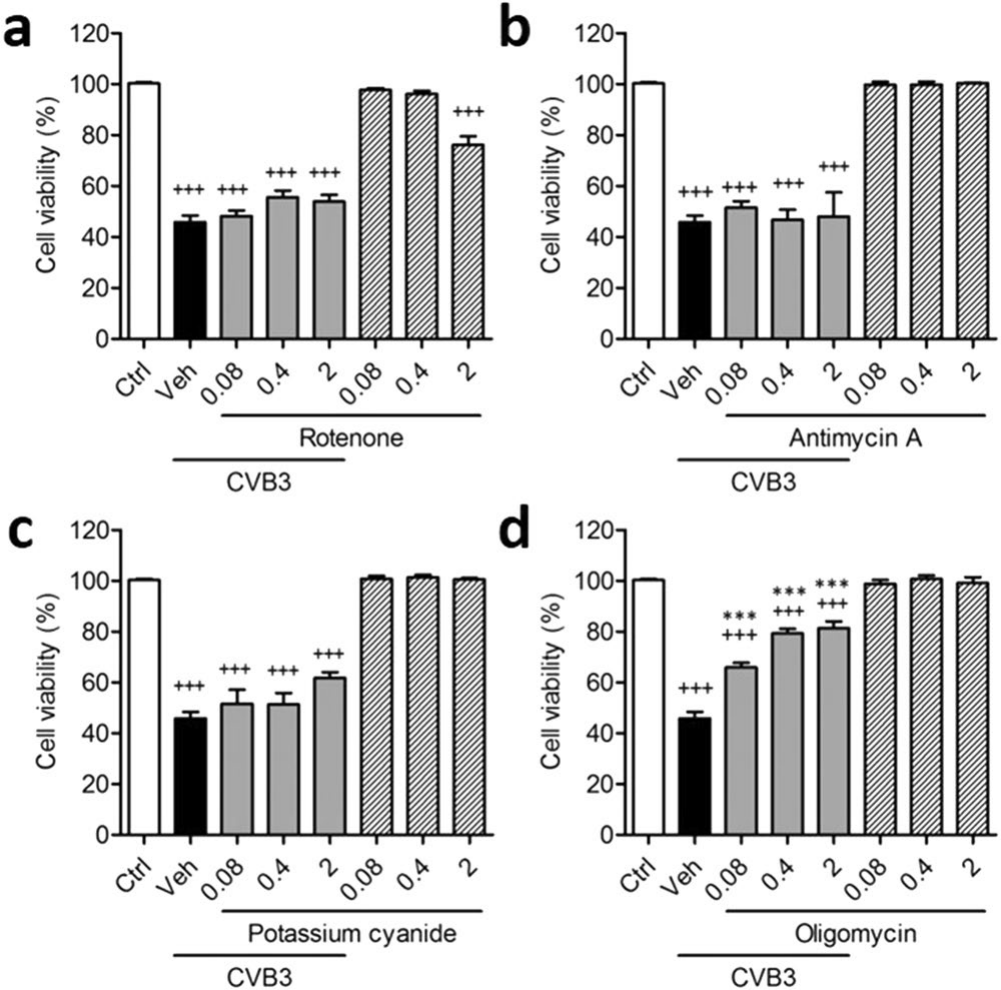
Antiviral activity of mitochondrial electron transport chain complex inhibitors against coxsackievirus B3 *in vitro*. Electron transport chain complex inhibitors rotenone (complex I inhibitor), antimycin A (complex III inhibitor), potassium cyanide (complex IV inhibitor), and oligomycin (complex V inhibitor) were evaluated for their antiviral activity against coxsackievirus B3 (CVB3) in Vero cells. Vero cells were infected with CVB3 in the presence or absence of 0.08, 0.4, and 2 µg/mL of (**a**) rotenone, (**b**) antimycin A, (**c**) potassium cyanide, or (**d**) oligomycin for 48 h. Data are presented as means ± SEM of 3 independent experiments. ^+++^*P* < 0.001 for comparison with non-infected control group (Ctrl); ***P < 0.001 for comparison with CVB3-infected vehicle group (Veh), Bonferroni’s multiple comparison test (ANOVA).

Man B is known to inhibit complex I and V of mitochondrial respiratory chain complex I^29^. Thus, we hypothesised that the antiviral activity of Man B toward CVB3 might be associated with mROS production by inhibition of the mitochondrial respiratory chain complex. Thus, we assessed whether other electron transport chain complex inhibitors, including rotenone (complex I inhibitor), antimycin A (complex III inhibitor), potassium cyanide (complex IV inhibitor), or oligomycin (complex V inhibitor), might have antiviral activity toward CVB3 *in vitro*. Rotenone showed about 20% cytotoxicity at a concentration of 2 μg/mL; however, antimycin A, potassium cyanide, and oligomycin did not reveal any cytotoxicity at the same concentration. Furthermore, rotenone, antimycin A, and potassium cyanide did not show antiviral activity at concentrations of 0.08, 0.4, and 2 μg/mL. Only oligomycin showed significant antiviral activity by preventing CVB3-induced cytotoxicity of Vero cells at each concentration (Fig. 4a–d). In addition, co-treatment with Man B and oligomycin did not result in additional antiviral activity compared with that of Man B or oligomycin alone (Supplementary Fig. S6). According to these data, the antiviral activity of Man B could be associated with the inhibition of electron transfer chain complex V but not complex I.

### Man B triggered the STING/TBK-1/IRF3 signalling pathway in CVB3-infected cells

**Figure 5:**
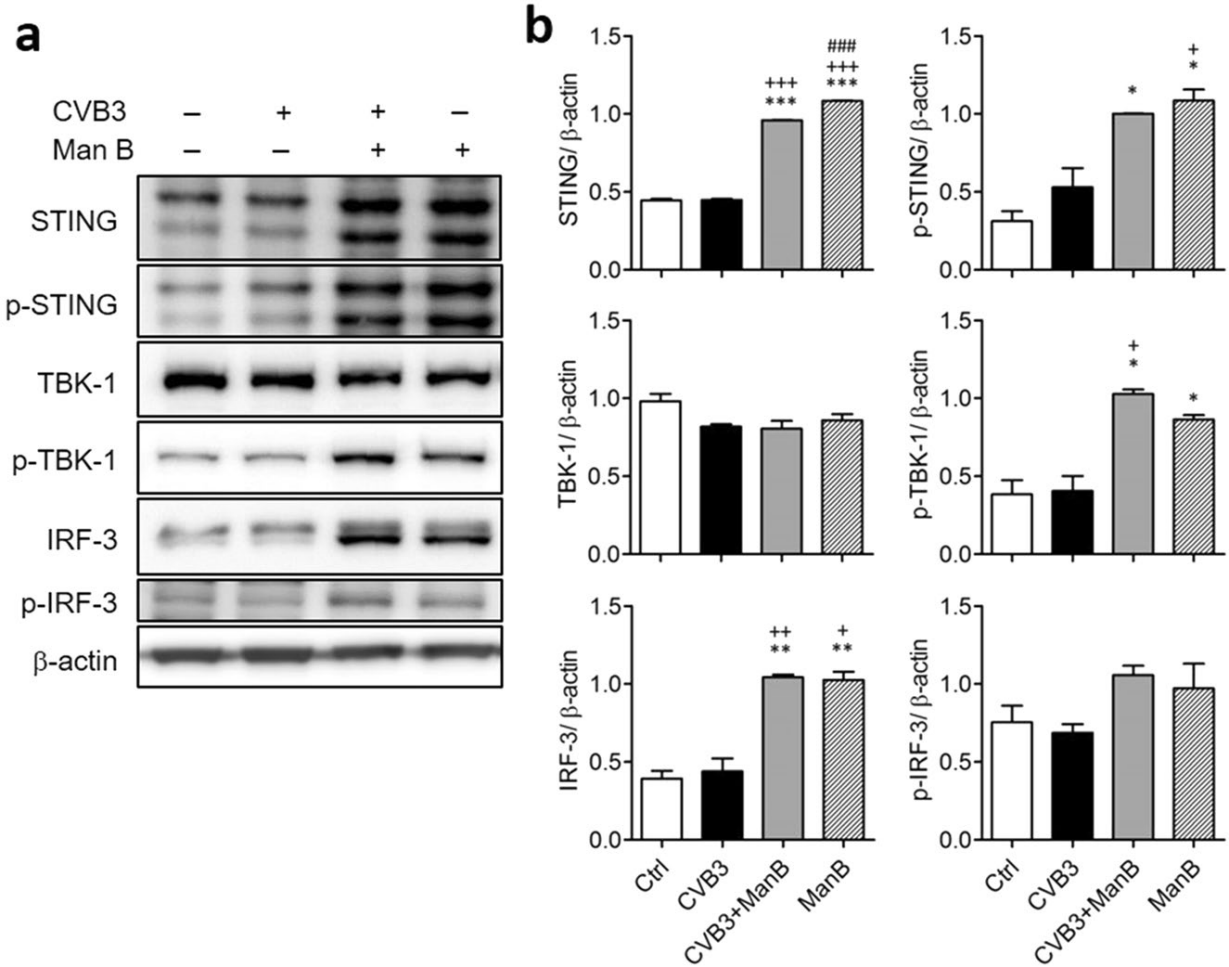
Manassantin B induced STING/TBK-1 signalling in coxsackievirus B3-infected cells. (**a**) Representative immunoblot of STING, p-STING, TBK-1, p-TBK-1, IRF-3, p-IRF-3, and β-actin to evaluate activation of their expression in coxsackievirus B3 (CVB3)-infected Vero cells or uninfected cells after treatment with manassantin B (Man B) (2 µg/mL). The samples were derived from the same experiment, and gels/blots were processed in parallel. (**b**) Density of western blot bands was calculated as the relative expression of STING, p-STING, TBK-1, p-TBK-1, IRF-3, and p-IRF-3. Each band was normalised by the density of the β-actin band. Data are presented as means ± SEM of 3 independent experiments. *P < 0.05, **P < 0.01, ***P < 0.001 for comparison with non-infected control group (Ctrl); ^+^*P* < 0.05, ^++^*P* < 0.01, ^+++^*P* < 0.001 for comparison with CVB3-infected vehicle group; ^*###*^*P* < 0.001 for comparison with CVB3-infected Man B group, Bonferroni’s multiple comparison test (ANOVA).

Cytosolic mtDNA can be recognised by cyclic GMP–AMP synthase (cGAS), thus possibly resulting in activation of the STING/TBK-1/IRF3 pathway, which is critical for the expression of antiviral genes^30,31^. Immunoblotting analysis of STING, p-STING, TBK-1, p-TBK-1, IRF-3, and p-IRF-3 in comparison with β-actin suggested that the expression levels of STING and IRF-3 were significantly increased by Man B treatment in both control and CVB3-infected cells. Furthermore, the levels of STING and TBK-1 phosphorylation were highly increased, suggesting that Man B activated the STING/TBK-1/IRF3 pathway for successful antiviral defence against CVB3 in infected cells (Fig. 5a,b).

## Discussion

Although a variety of pharmacological activities associated with *S. chinensis* have been demonstrated^13,14,32^, antiviral activities of *S. chinensis* toward coxsackieviruses have not been reported. In this study, we isolated and characterised Man B from *S. chinensis* and demonstrated its strongly antiviral activity against CVB3.

Currently, the prevention and treatment of coxsackievirus infection are limited. A vaccine to prevent coxsackievirus infection is difficult to produce because there are many immunologically non-cross-reactive serotypes. Ribavirin, a broad-spectrum nucleoside analogue, exhibited expected antiviral action. This antiviral drug was previously shown to have an inhibitory effect on many DNA and RNA viruses in cell culture^33^. However, ribavirin showed only a marginal effect on CVB3 infection of Vero cells. Further, viruses that had acquired resistance to ribavirin were isolated from several virus populations and observed in some patients^34^. Therefore, the existence of ribavirin-resistant viruses suggests that development of a novel antiviral drug may be especially important to treat coxsackievirus infection.

In the current study, we found that Man B exhibited strong antiviral activity against coxsackieviruses with IC_50_ values ranging from 0.88 to 8.20 µg/mL. The cytotoxicity of Man B against Vero cells was not observed up to 10 µg/mL, suggesting that Man B is not cytotoxic below 10 µg/mL. Although significant toxicity was not found in mice after four consecutive daily treatments of Man B (2.5 mg/kg/day), the toxicity should be carefully tested in animal models to ensure that Man B is safe *in vivo*.

A recent study showed that CVB3 infection in resistant C57BL/6 mice led to rapid elimination of virus in the heart as a result of enhanced mROS, which might be due to alteration of the mitochondrial respiratory chain^35^. However, at the cellular level, C3 (pro) cysteine protease of CVB3 cleaved MAVS and TRIF to inhibit type I IFN cytokine production, and consequently, CVB3 infection did not induce a significant type I IFN response in HEK293, HeLa, and Caco-2 cells^36,37^. MAVS, which is localised in the mitochondrial membrane, is a downstream adaptor molecule that transfers signals for activation of RIG-I, and several viruses including CVB3 employ strategies to disrupt MAVS-dependent antiviral pathways^37^.

Several studies have shown that ROS generation is linked to the regulation of antiviral signalling as well as mitochondrial homeostasis and apoptotic cell death^38^. RIG-I-like receptor (RLR) signalling for the recognition of viral infection is dependent on mROS level^39^. Interestingly, several mitochondrial resident molecules including MAVS might converge with mROS levels for successful IFN signalling^40,41^. Indeed, a recent study showed that one of the mitochondrial electron transport molecules composing complex IV, cytochrome C oxidase complex subunit 5B (COX5B), physically interacts with MAVS and inhibits MAVS signalling by repressing mROS. In addition, COX5B appears to coordinate with autophagy to negatively regulate MAVS-dependent antiviral signalling^38^.

Man B was identified as a specific inhibitor of mitochondrial respiratory chain complex I through phenotypic screening using zebrafish embryos^29^. Biochemical assay suggested that the IC_50_ value of Man B for complex I inhibition was less than 25 nM and that Man B shares some mechanistic characteristics with rotenone^29^. Although treatment of cells with rotenone induced apoptotic cell death by inhibiting mitochondrial complex I, HIV-1-infected cells were insensitive to rotenone-induced cell death, suggesting that mitochondrial complex I might be downregulated, or mROS generation might be blocked by some viral infection such as HIV-1 infection^42^. Likewise, we found that treatment of Vero cells with CVB3 slightly but not significantly inhibited mROS generation as compared with that of control cells treated with vehicle only (Fig. 3b).

**Figure 6:**
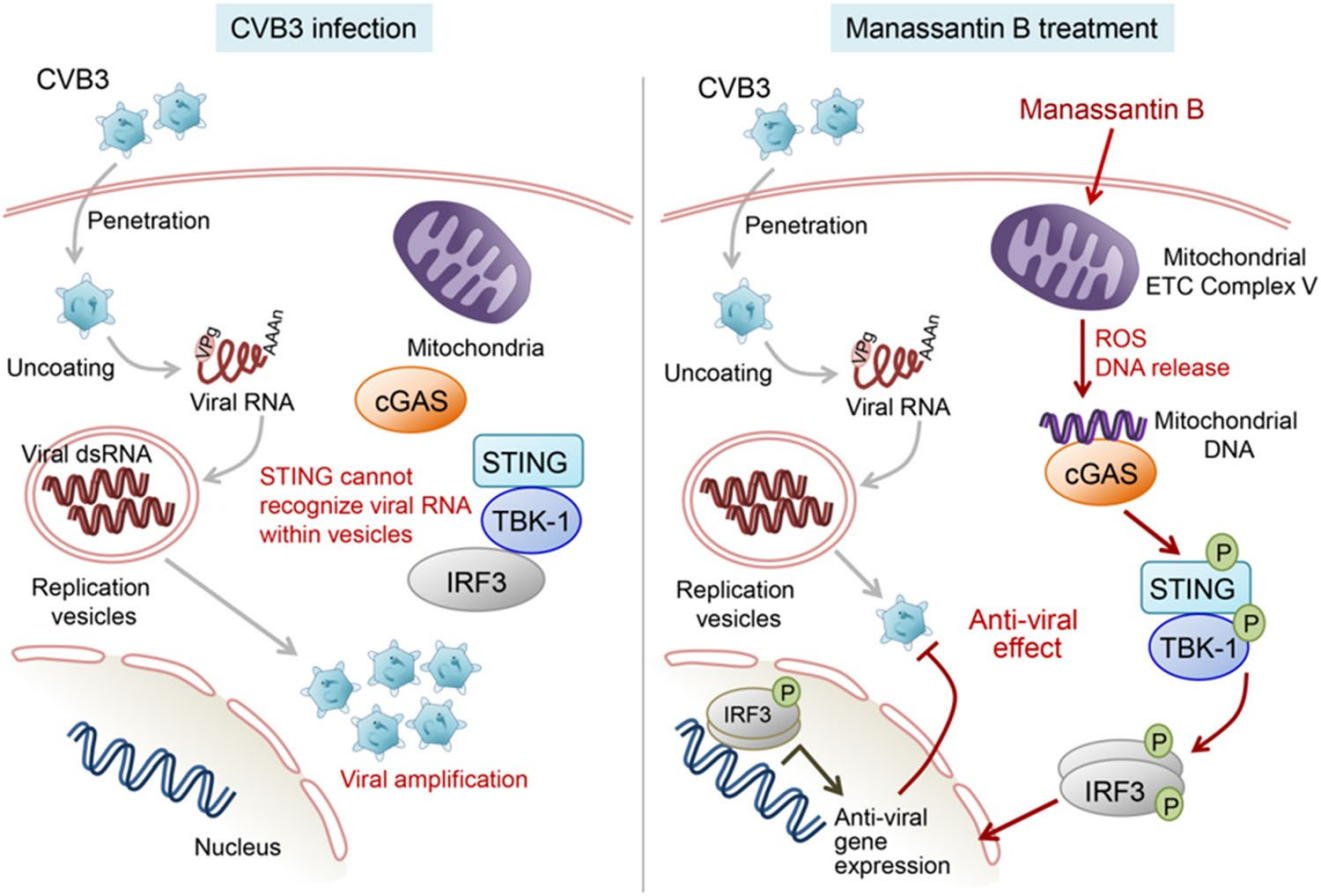
Proposed mechanism for antiviral activity of manassantin B toward coxsackievirus B3. Once coxsackievirus B3 (CVB3) infects host cells, virus particles are uncoated from their capsid protein and subsequently release viral RNA into the cytosol. Because viral replication occurs inside replication vesicles such as intracellular organelles, host pattern-associated molecular pattern sensor proteins, such as cGAS and MAVS, cannot adequately recognise their counterpart ligand. When manassantin B is administered to CVB3-infected cells, it can suppress mitochondrial electron transport chain (ETC) complex 1 and 5. Suppressed ETC results in increased mitochondrial ROS production and shutdown of mitochondrial function. In addition, mitochondria suppressed by manassantin B release their own DNA, which may be recognised by cGAS. Mitochondrial DNA-recognised cGAS synthesises cGAMP, which serves as a secondary messenger, by conjugating ATP and GTP. cGAMP binds with STING, resulting in STING activation and TBK-1 recruitment. Phosphorylated TBK-1 phosphorylates IRF-3, which dimerises, is imported into the nucleus, and serves as a transcription factor for antiviral gene expression. Finally, viral replication can be suppressed by antiviral gene expression, which is activated by manassantin B.

Although CVB3 infection alone did not induce mROS production in Vero cells, treatment with Man B significantly increased mROS production. Since mROS is closely associated with mitochondrial damage^43^, we presume that Man B can induce mtDNA release into the cytosol, which may activate cGAS to generate cyclic dinucleotide cyclic-GMP–AMP (cGAMP) as a secondary messenger^44^. The increase in cGAMP triggers kinase activity of STING and transduces p-IRF3/IRF3 signalling to switch on antiviral transcription in nuclei of infected cells^45^. In our study, CVB3-infected cells did not activate IRF-3 phosphorylation; however, Man B treatment of those cells significantly induced the phosphorylation of TBK-1 and IRF-3 to activate antiviral gene expression, which was inhibited by CVB3 (Fig. 6).

In the current study, we showed that Man B inhibited the replication of CVB3 by activating the STING/TBK-1/IRF3 pathway as well as increasing mitochondrial ROS. Recently, cGAS cytosolic DNA was implicated in the viral sensing pathways including the STING/TBK-1/IRF3 pathway via G3BP1^46,47^. Because G3BP1–eIF4G1, which is important for stress granule formation, was reported as a target of picornavirus proteases^48,49^, it would be interesting to evaluate whether the antiviral activity of Man B is associated with the regulation of G3BP1.

Thus, our results suggest that the antiviral activity induced by Man B treatment was associated with production of mROS and activation of type I IFN signalling through activation of the cGAS/STING/TBK-1/IRF3 pathway. In the future, we will continue evaluating whether such strategy is applicable for the development of antiviral drugs and identifying antiviral drug candidates through screening of compounds that induce mitochondrial damage.

## Methods

### Extraction and isolation of active compounds from *S. chinensis* Baill

Aerial portions of *S. chinensis* Baill were purchased at Gyeongsangnamdo Agricultural Research & Extension Services, Korea, in November 2009. The dried, ground plants (1.2 kg) were finely powdered with a blender, after which the powder was macerated in 3 L of methanol at room temperature for 3 days. Then, the macerate was filtered (Whatman No. 2), and the process was repeated three times. The combined filtrates were evaporated using a 40 °C water bath to produce (yield) 84.2 g of a dark green residue. The methanol extract (84.2 g) was dissolved in 1 L of water and then separated with *n*-hexane, ethyl acetate, and *n*-butanol, respectively. The ethyl acetate-soluble extract was evaporated under reduced pressure to yield 30.07 g of residue. Next, the ethyl acetate-soluble extract (30.07 g) was subjected to C_18_ column chromatography (40–63 μm, 300 g; Merck, Kenilworth, NJ, USA) and eluted with a gradient consisting of methanol:water (2:8, 4:6, 6:4, 8:2, 10:0; 2 × 500 mL each). Fractions of similar pattern were pooled to produce 10 fractions (Fr.1–Fr.10) on the basis of thin layer chromatography analysis. Based on the bioassay-guided fractionation, Fr.10 showed the most potent inhibitory effects against CVB3 (Supplementary Tables S1 and S2). Furthermore, Fr.10 was purified by preparative reversed-phase HPLC using a gradient of 30%–100% acetonitrile in water (Capcell Pak C18 UG120, 250 × 10 mm, 10 μm; Shisheido, Tokyo, Japan) to obtain Man B. The structures of active compounds were determined by spectroscopic analysis, including EI-MS, ^1^H-NMR, and ^13^C-NMR (Supplementary Fig. S1).

### Cell lines, viruses, and reagents

CVB3 (ATCC VR-30) was obtained from American Type Culture Collection (Manassas, VA, USA) and propagated at 37 °C in Vero cells maintained in minimal essential medium supplemented with 10% (v/v) foetal bovine serum (FBS) and 1% (v/v) antibiotic–antimycotic solution. Gibco^®^ antibiotic–antimycotic solution, trypsin-ethylenediamine tetraacetic acid, FBS, and minimal essential medium were purchased from Life Technologies (Carlsbad, CA, USA), and Falcon™ tissue culture plates were purchased from BD Biosciences (San Jose, CA, USA). Rotenone, antimycin A, potassium cyanide, and oligomycin were purchased from Sigma–Aldrich (St. Louis, MO, USA), and ribavirin was obtained from Duchefa Biochemie (Haarlem, The Netherlands).

### Antiviral activity assay

The day before infection, 3 × 10^4^ Vero cells/well were seeded onto a 96-well culture plate. The next day, the culture medium was aspirated, and the cells were washed with 1× PBS. Then, 0.09 mL of the diluted virus suspension containing the virus stock at a multiplicity of infection (MOI) of 0.8 with 1% FBS added to produce an appropriate cytopathic effect (CPE) within 2 days after infection, followed by the addition of 0.01 mL of test compound. The test compounds were prepared by a 10-fold dilution scheme. The antiviral activity of each test compound was determined with four concentrations ranging from 0.1 μg/mL to 10 μg/mL. We used three wells each for virus controls (virus-infected, non-drug-treated cells) and cell controls (non-infected, non-drug-treated cells). The 96-well culture plates were incubated at 37 °C in 5% CO_2_ for 2–3 days until 70%–80% CPE. To prevent false-positive results due to the presence of dead cells in the sulforhodamine B (SRB) assay, we discarded the supernatant and thoroughly washed the wells with PBS twice at the end of culture. Then, cells were fixed with ice-cold 70% acetone (100 μL/well) for 30 min at −20 °C and stained with 0.4% SRB in 1% acetic acid. SRB-stained cells were solubilised with 10 mM unbuffered Tris base solution, and the absorbance was measured at 562 nm using a SpectraMax® i3 microplate reader (Molecular Devices, Palo Alto, CA, USA) with a reference absorbance of 620 nm. The results were then transformed into percentages of the controls, and the percent protection achieved by the test compound in the virus-infected cells was calculated using the following formula: [(OD_t_)_virus_ − (OD_c_)_virus_] ÷ [(OD_c_)_mock_ − (OD_c_)_virus_] × 100%, where (OD_t_)_virus_ is the optical density measured with a given concentration of the test compound in virus-infected cells; (OD_c_)_virus_ is the optical density of the non-drug-treated, virus-infected control cells; and (OD_c_)_mock_ is the optical density.

### PCR analysis

Vero cells in culture plates were tested when confluent. The culture medium was removed, and the cells were washed with PBS. Then, 90 μL of diluted virus suspension and 10 μL of medium supplemented with 1% FBS containing active compounds were added. The 96-well culture plates were incubated at 37 °C in 5% CO_2_ for 48–72 h until 70%–80% CPE. Cells exhibiting 70% CPE were frozen and thawed three times, and viral RNA was extracted from the infected cells using the QIAamp Viral RNA Mini kit (Qiagen, Hilden, Germany)^50^. cDNA was generated in a 20-μL reaction for 30 min at 42 °C using 1 μg RNA, random primers, and SuperScript II reverse transcriptase (Invitrogen) according to the manufacturer’s instructions^50^. RT-PCR was performed with a GeneAmp PCR System 2700 (Perkin-Elmer/Cetus, Norwalk, CT, USA) using primer sets specific for the 5′ NCR of the enterovirus and β-actin^51^ of Vero cells. Briefly, a 50-μL reaction mixture containing 0.2 μM of ENT-F and ENT-R^52^ or β-actin primers^53^, 2 U of Taq DNA polymerase (Promega, Madison, WI, USA), 100 μM dNTPs, and 2 μM MgCl_2_ was subjected to 35 cycles of 94 °C for 1 min, 52 °C for 1 min, and 72 °C for 1 min. The final extension step was extended to 72 °C for 7 min^54^. PCR-amplified products were separated on 1.5% agarose gels containing 0.1 μg/mL ethidium bromide and visualised under UV light.

Quantitative real-time PCR was conducted with 1 μL of cDNA. A customised AccuPower^®^ Enterovirus Real Time RT-PCR Kit (Bioneer Corp., Daejeon, South Korea) and QuantStudio 5 system (Thermo Fisher Scientific, Waltham, MA, USA) were used for quantification under the following conditions: 10 min at 95 °C for initial denaturation, followed by 45 cycles of amplification with denaturation at 95 °C for 15 s and annealling/extension at 55 °C for 30 s. A standard curve was generated using 10-fold serial dilutions (10^9^ to 10^3^ copies/test) of *in vitro*-synthesised RNA strands.

### Western blotting

Rabbit anti-cytoskeletal actin (Bethyl Laboratories, Montgomery, TX, USA), mouse anti-CVB3 VP1 (Dako, Copenhagen, Denmark), mouse anti-α tubulin (Santa Cruz Biotechnology, Dallas, TX, USA), rabbit anti-STING, rabbit anti-phospho-STING (Ser336), rabbit anti-TBK-1, rabbit anti-phospho-TBK-1 (Ser172), rabbit anti-IRF-3, and rabbit anti-phospho-IRF-3 (Ser396) (all from Cell Signaling Technologies, Danvers, MA, USA) antibodies were used. The enhanced chemiluminescence substrate femto LUCENT™ PLUS-HRP (G-Biosciences, St. Louis, MO, USA) was applied, and images of bands were captured using an Image Quant™ LAS 4000 Mini system (GE Healthcare Life Sciences, Little Chalfont, UK). Quantification of band densities was performed using ImageJ software (NIH, Bethesda, MD, USA).

### mROS measurement

Vero cells (1×10^6^) were seeded onto a 6-well plate. The next day, the supernatant of each well was replaced with viral infection media or fresh media with Man B. After 4 h, cells were harvested by trypsinisation and stained with MitoSox Red (Invitrogen) mitochondrial superoxide indicator according to the manufacturer’s guideline. Stained cells were analysed via FACSVerse (BD Biosciences) without the formalin fixation step.

### mtDNA detection in cytosolic fraction

After no treatment, CBV3 infection, CBV3 infection and Man B treatment, or Man B treatment only, 2×10^6^ Vero cells were harvested. Each cell suspension was equally divided into two aliquots. One aliquot was used for extraction of the cytosolic fraction with the Mitochondria/Cytosol fractionation kit (Enzo Life Sciences, Farmingdale, NY, USA). The other aliquot was used for genomic DNA extraction via the QIAamp DNA Blood Mini Kit (Qiagen). To quantify mtDNA in the cytosolic fraction, real-time PCR was performed with cytochrome C oxidase primers. Cytosolic cytochrome C oxidase amplification was normalised by β-actin amplification of whole genomic DNA.

### Animal model

Female BALB/c mice aged 4 weeks were purchased from SPL Laboratory Animal Company (Koatech Bio, Pyeongtaek, Korea). All mice were maintained under specific pathogen-free conditions for 1 week in the experimental facilities at Kangwon National University (Chuncheon, Korea), where they received sterilised food and water *ad libitum* and were housed at 20–22 °C with a 12-hours light/dark cycle. All animal experiments were performed in accordance with guidelines set and approved by the Institutional Animal Care and Use Committees of Kangwon National University (KW-161101-2). Five-week-old female BALB/c mice were intraperitoneally inoculated with 1 × 10^6^ pfu/mouse of CVB3. BALB/C mice infected with CVB3 were orally administered Man B (2.5 mg/kg/day) or ribavirin (10 mg/kg/day) for 4 days^27^.

### Cytokine and chemokine assay

We confirmed the level of cytokines and chemokines with ELISA kits for TNF-α, IL-6, IFN-γ, CCL2 (MCP1) (all from eBioscience, San Diego, CA, USA), and KC (CXCL1) (R&D Systems, Minneapolis, MN, USA). Serum was obtained as previously described^55^. Lungs were obtained from mice infected with CVB3, and the same amount of lung tissue was homogenised to obtain supernatants. The cytokine and chemokine levels in lung supernatant were evaluated according to the manufacturer’s instructions^25^. The absorbance was then read at 450 nm using a SpectraMax 340 (Molecular Devices).

### Statistical analysis

We used Student’s *t*-test to compare the differences between two groups. To compare multiple groups, we performed one-way ANOVA followed by Bonferroni’s multiple comparison test. Values of *P* < 0.05 were considered significant at a 95% confidence interval.

## Supplementary information

Supplementary files
