# Supplementary material for: Manassantin B shows antiviral activity against coxsackievirus B3 infection by activation of the STING/TBK-1/IRF3 signalling pathway
Source: Sci Rep. 2019 Jun 28;9:9413. doi: 10.1038/s41598-019-45868-8 (PMC6599049; doi:10.1038/s41598-019-45868-8)
Supplement: Supplementary file 1 — Supplementary files [file 41598_2019_45868_MOESM1_ESM.pdf]

Manassantin B shows antiviral activity against coxsackievirus B3 infection by activation of the STING/TBK-1/IRF3 signalling pathway

Jae-Hyoung Song[1,†], Jae-Hee Ahn[1,†], Seong-Ryeol Kim[1], Sungchan Cho[3], Eun-Hye Hong[1], Bo-Eun Kwon[1], Dong-eun Kim[3], Miri Choi[3], Hwa-Jung Choi[4], Younggil Cha[1], Sun-Young Chang[2,*], Hyun-Jeong Ko[1,*]

Supplementary Table S1. Antiviral activity of *Saururus chinensis* Baill

Botanical	Solvent	CC_{50}[a]	IC_{50}[b]	TI[c]
Saururus chinensis Baill	Hexane	66.03	7.89 ± 1.21	8.36
Saururus chinensis Baill	Ethyl acetate	>100	4.59 ± 1.66	21.78
Saururus chinensis Baill	Butanol	>100	43.81 ± 8.47	2.28

Results are presented as mean IC_{50} values obtained from three independent experiments carried out in triplicate ± S.D.

[a] Concentration required to reduce cell growth by 50% (μg/mL)

[b] Concentration required to inhibit virus-induced CPE by 50% (μg/mL)

[c] Therapeutic index = CC_{50} / IC_{50}

Supplementary Table S2. Antiviral activity of C18 column chromatography fractions against CVB3

Fraction	CC_{50}[a]	IC_{50}[b]	TI[c]
1	>100	ND[d]	-
2	>100	ND[d]	-
3	>100	ND[d]	-
4	>100	10.84±5.87	9.23
5	>100	47.42±16.20	2.11
6	>100	28.48±14.66	3.51
7	>100	5.68±0.67	17.61
8	62	6.78±1.55	9.14
9	>100	22.88±23.62	4.37
10	>100	4.79±0.24	20.88

Results are presented as mean IC_{50} values obtained from three independent experiments carried out in triplicate ± S.D.

[a] Concentration required to reduce cell growth by 50% (μg/mL)

[b] Concentration required to inhibit virus-induced CPE by 50% (μg/mL)

[c] Therapeutic index = CC_{50} / IC_{50}

[d] The IC_{50} value was not determined because of a maximum inhibition rate of <50%.

Supplementary Table S3. **^{1}H- (400 MHz) and ^{13}C-NMR (100 MHz) chemical shifts of manassantin B (in $CDCl_3$, δ in ppm)**

Position	^{1}H-NMR[a]	^{13}C-NMR
1		136.5
2	a	110.6
3		146.3
4		150.5
5	a	108.5
6	a	118.6
7	5.46 (1H, d, *J* = 6 Hz)	83.3
8	2.28 (1H, m)	44.2
9	0.71 (3H, d, *J* = 6.0 Hz)	14.8
O-CH_3-3	3.93 (3H, s)	55.8
1'		136.5
2'	a	110.9
3'		146.4
4'		150.5
5'	a	107.5
6'	a	118.6
7'	5.46 (1H, d, *J* = 6 Hz)	83.3
8'	2.28 (1H, m)	44.2
9'	0.71 (3H, d, *J* = 6.0 Hz)	14.8
O-CH_3-3'		55.8
1''		110.9
2''	a	132.5
3''		147.3
4''		148.8
5''	a	118.6
6''	a	119.9
7''	4.65 (1H, d, *J* = 8.4 Hz)	78.4
8''	4.10 (1H, m)	84.0
9''	1.17 (3H, d, *J* = 6.2 Hz)	16.9
O-CH_3-3''	3.89 (3H, s)	55.8
O-CH_3-4''	3.88 (3H, s)	55.8
1'''		110.8
2'''	a	133.9
3'''		149.0
4'''		147.7
5'''	a	118.7
6'''	a	121.0
7'''	4.61 (1H, d, *J* = 8.8 Hz)	78.4
8'''	4.10 (1H, m)	84.0
9'''	1.14 (3H, d, *J* = 6.2 Hz)	16.9
O-CH_2-O	5.94 (2H, s)	101.0

[a] 6.76–7.00 (12H, m, aromatic protons)

Supplementary Table S4. Antiviral activity of manassantin B against coxsackieviruses in Vero cells

Coxsackievirus	Manassantin B		
	CC_{50}[a]	IC_{50}[b]	TI[c]
B1	>10	6.17 ± 0.91	1.62
B2	>10	4.81 ± 1.34	2.07
B3	>10	0.88 ± 0.05	11.3
B4	>10	6.51 ± 0.48	1.53
B5	>10	8.20 ± 0.98	1.21
B6	>10	7.72 ± 0.47	1.29

Results are presented as mean IC_{50} values obtained from three independent experiments carried out in triplicate ± S.D.

[a] Concentration required to reduce cell growth by 50% (μg/mL)

[b] Concentration required to inhibit virus-induced CPE by 50% (μg/mL)

[c] Therapeutic index = CC_{50} / IC_{50}

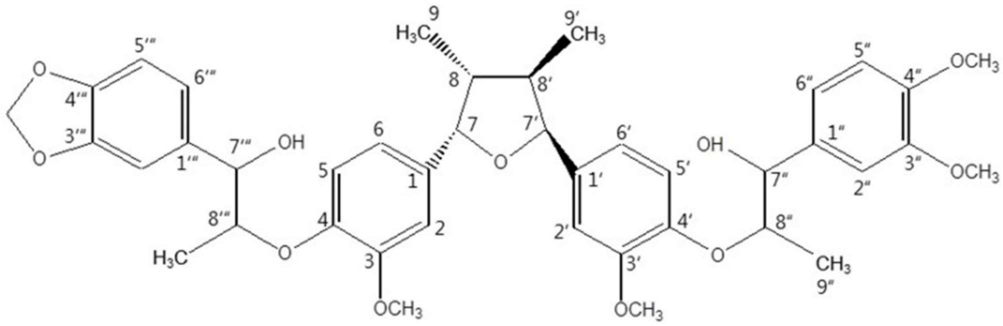

Supplementary Figure S1. Structure of Man B.

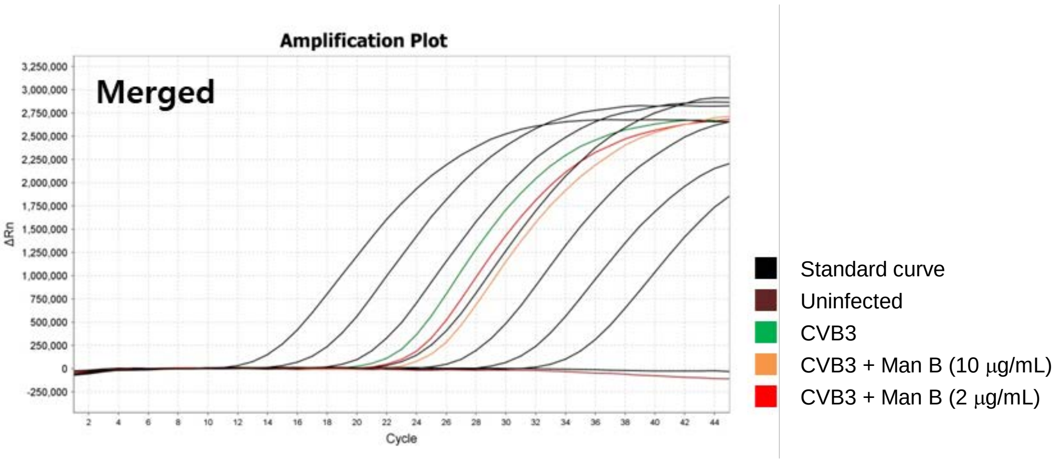

Supplementary Figure S2. Quantitative real-time PCR to determine the copy numbers of CVB3 viral RNA in Vero cells treated with different concentrations of Man B.

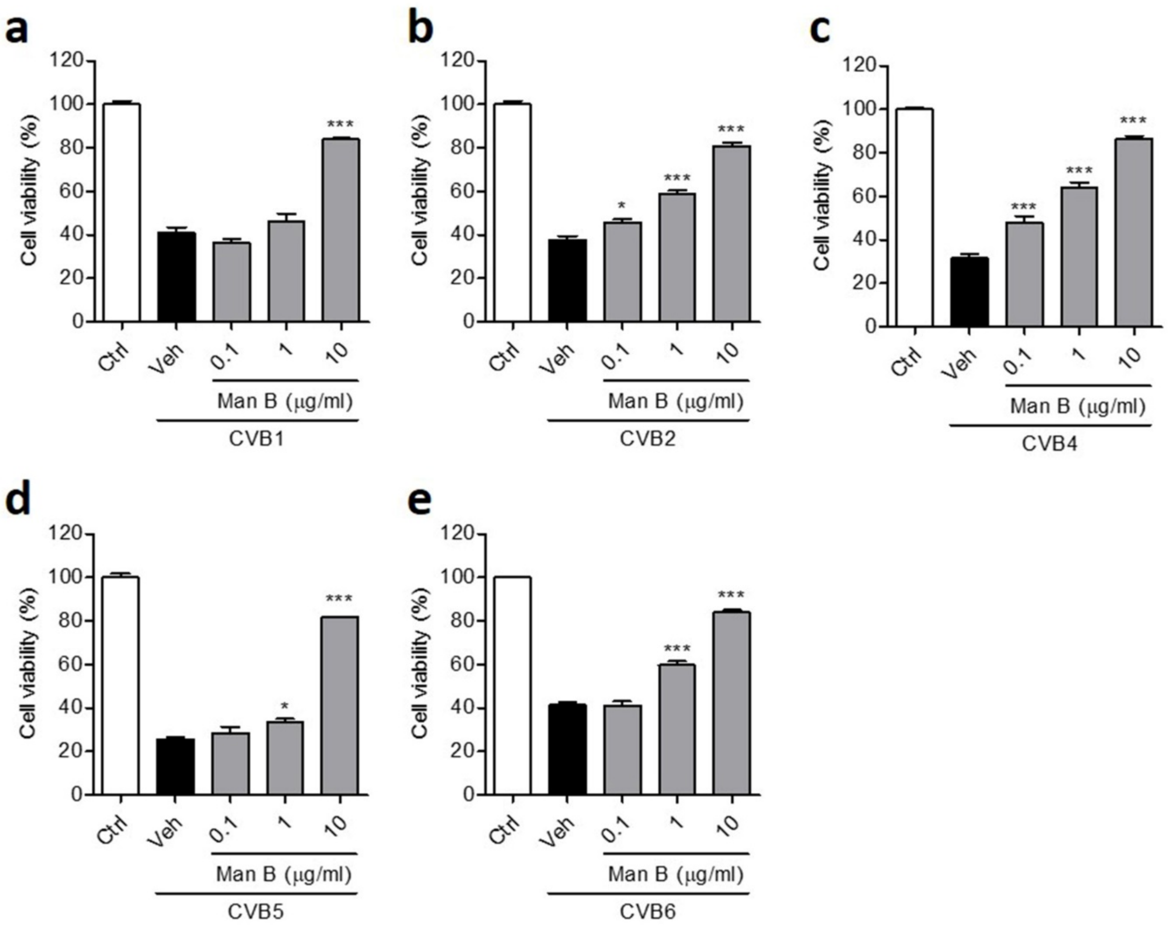

Supplementary Figure S3. Antiviral activity of Man B against other group B coxsackieviruses including (a) CVB1, (b) CVB2, (c) CVB4, (d) CVB5, and (e) CVB6 *in vitro*.

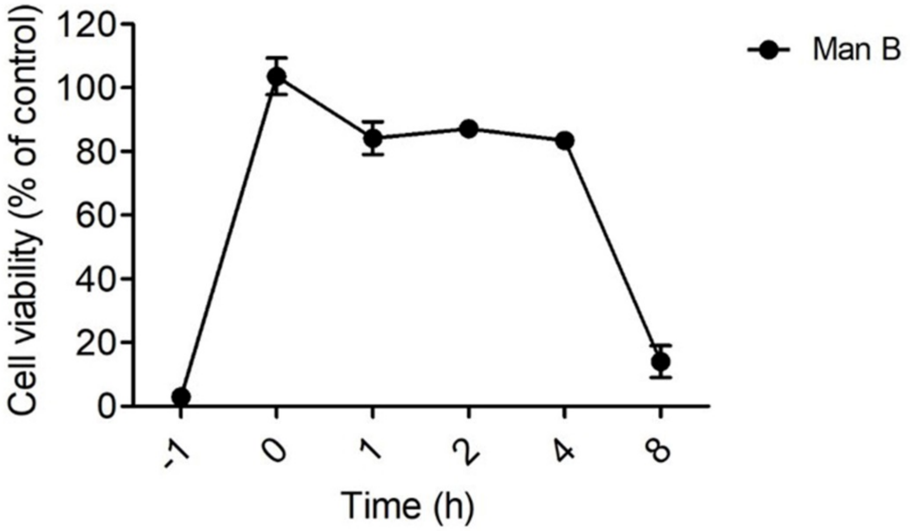

Supplementary Figure S4. Cell viability of Vero cells treated with Man B (10 μg/ml) before, during, and after CVB3 viral infection determined by SRB assay. Cell viability was analyzed 48 h postinfection.

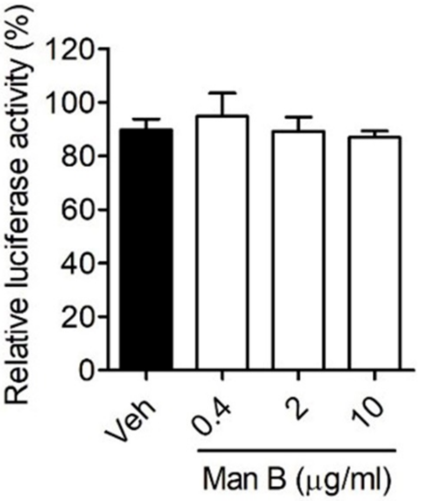

Supplementary Figure S5. Dual-luciferase reporter assay to determine CVB3 IRES-dependent translation. Twenty-four hours after treatment with Man B, the intensity of luciferase activity was assessed in 293T cells. The luciferase activity of DMSO-treated cells was considered to be 100%.

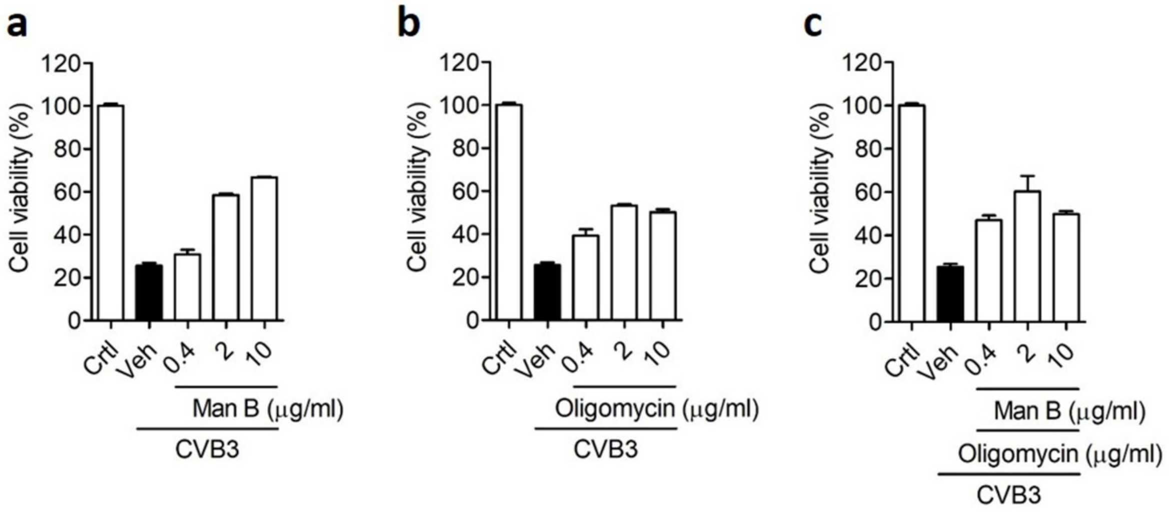

Supplementary Figure S6. Cell viability of Vero cells determined using SRB assay. Cells infected with CVB3 were incubated with (a) Man B, (b) oligomycin, or (c) Man B and oligomycin at the indicated concentrations for 48 h.
